# Hepatosplenic Sarcoidosis: Contrast-Enhanced Ultrasound Findings and Implications for Clinical Practice

**DOI:** 10.1155/2014/926203

**Published:** 2014-08-18

**Authors:** Claudio Tana, Christoph F. Dietrich, Cosima Schiavone

**Affiliations:** ^1^Unit of Internistic Ultrasound, Department of Medicine and Science of Aging, “G. d'Annunzio” University, Via dei Vestini 29, 66100 Chieti, Italy; ^2^Medizinische Klinik 2, Caritas-Krankenhaus, Uhlandstraße 7, 97980 Bad Mergentheim, Germany

## Abstract

Sarcoidosis is a complex granulomatous disease that affects virtually every organ and tissue, with a prevalence that varies significantly among the sites involved. The role of conventional imaging, such as computed tomography and magnetic resonance imaging, in the assessment of hepatosplenic sarcoidosis is well established by revealing organ enlargement, multiple discrete nodules, and lymphadenopathy. In this review, we aim to describe contrast-enhanced ultrasound (CEUS) findings in liver and spleen involvement by sarcoidosis, reporting evidence from the literature and cases from our experience, after a brief update on safety profile, cost-effectiveness, and clinical indications of this novel technique. Furthermore, we highlight potential advantages of CEUS in assessing hepatosplenic sarcoidosis that may be useful in the clinical practice.

## 1. Introduction

Sarcoidosis is a complex granulomatous disease that virtually affects every organ and tissue, with a prevalence that varies significantly among the sites involved. However, it affects most often compartments such as lungs and mediastinal lymph nodes, manifesting as a pulmonary restrictive disorder in up to 65% of patients, and with pulmonary fibrosis in 20–25% of them [1–3]. The value of mediastinal ultrasound in patients with sarcoidosis has been recently shown [4]. The high prevalence of pulmonary disease could be associated with the primary activation of alveolar macrophages by inhaled exogenous agents, such as inorganic particles, insecticides used at work, and those from exposition to moldy environments [1–3]. The formation of typical noncaseating granulomas represents the final product of an incomplete antigens degradation, associated with an exuberant macrophage and T- and B-cell activity due to prolonged antigenaemia [5, 6]. Also genetic factors (both HLA and non-HLA genes) have been associated with an increased risk of sarcoidosis, and the complex interaction between endogenous and exogenous agents may reflect the great variability of clinical manifestations [7].

Organs, such as liver and spleen, are less frequently affected than lungs, and their involvement often shows a benign course but portal hypertension and loss of liver function may occur [8–10]. However, a correct evaluation of these organs represents an important step in patients with sarcoidosis, before starting appropriate treatment.

## 2. Clinical and Laboratory Findings in Hepatosplenic Sarcoidosis: When to Perform Imaging Studies

Although there are no specific recommendations for hepatosplenic imaging studies in patients with sarcoidosis, it is widely accepted to perform exams such as unenhanced ultrasound, computed tomography (CT), and magnetic resonance imaging (MRI) when an increase of liver function tests is found or when abdominal symptoms, such as nausea, vomiting, and weight loss, are reported.

Liver can be affected in 10–25% of cases, but hepatic involvement is often oligo- or asymptomatic. Various degrees of dysfunction of liver function tests such as alanine aminotransferase (ALT), aspartate aminotransferase (AST), gamma-gt, and alkaline phosphatase can be observed [8]. In a recent study that comprises 837 patients with sarcoidosis, an increase of ALT and AST was found in up to 15% of cases [11]. Hepatic sarcoidosis can manifest with constitutional symptoms such as weight loss, anorexia, fever, and night sweats [1] or, less frequently, with symptoms related to chronic intrahepatic cholestasis, such as pruritus and jaundice. In these cases, laboratory tests reveal an increase of alkaline phosphatases and total and direct bilirubin [12]. Rarely, cholestasis is associated with common bile duct compression by a mass in the pancreatic head or by enlarged perihepatic lymph nodes [13]. Only few cases complicated by cirrhosis and portal hypertension have been reported in the literature, and they present with ascites and/or bleeding from rupture of gastroesophageal varices [14–16].

Splenic involvement is uncommon (5–10% of cases) and usually manifests with asymptomatic and mild splenomegaly. Rarely, the enlargement of the spleen is more pronounced with hypersplenism and pancytopenia [8, 14].

## 3. Conventional Imaging in the Assessment of Hepatosplenic Sarcoidosis

### 3.1. B-Mode and Color Doppler Ultrasound Findings

Hepatosplenic sarcoidosis is common in patients with systemic disease, but it is often underestimated on imaging techniques, in particular conventional ultrasound, because granulomatous inflammation of the liver and spleen can be minimal and/or manifest with nonspecific patterns. While granulomas have been found in 60–80% of liver biopsy specimen, sarcoid hepatic nodules are found on imaging in only 5% of cases [17].

**Figure 1:**
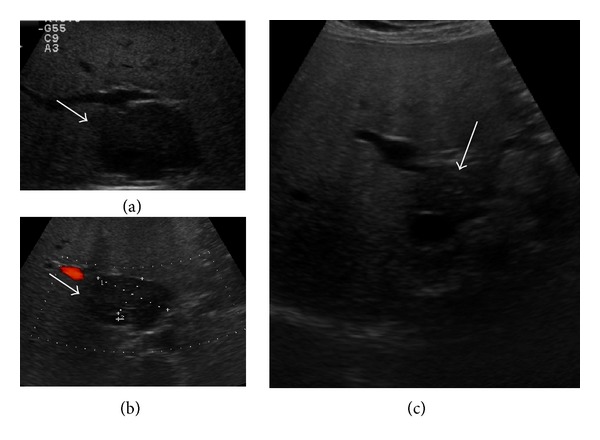
A 51-year-old woman with diagnosis of pulmonary sarcoidosis, who presented with dyspepsia. (a) B-mode US showed diffuse liver hyperechogenicity suggestive of fatty liver disease and a hypoechoic lesion in the hepatic segment I (arrow). The lesion was in close contiguity with inferior vena cava and had a maximum size of 51 mm. Imaging findings were suggestive of focal fatty sparing, but histopathological examination revealed noncaseating granulomas, suggesting liver involvement by sarcoidosis. Spleen was normal. (b) Color Doppler US showed no flow inside the nodule (arrow). (c) After six months of steroid therapy, the lesion was significantly reduced (arrow).

**Figure 2:**
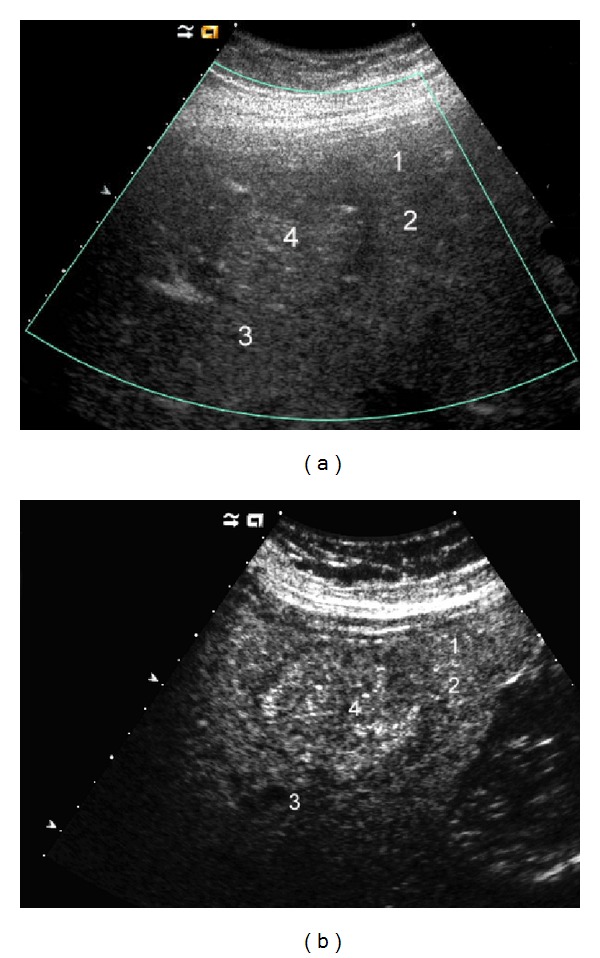
A 24-year-old female with histopathological diagnosis of hepatic sarcoidosis that resembled advanced stage of cirrhosis on US. (a) The liver was almost completely subverted by multiple more diffuse and also more circumscribed hypoisoechoic nodules (numbers 1 to 4); the lesions did not demonstrate vascularity on Color Doppler US. (b) Contrast-enhanced US in the late phase showed almost isoenhancing lesions.

The most common finding is represented by hepatomegaly with homogenous distribution of echoes and without evidence of prominent nodules. Sometimes, US can demonstrate an increase of parenchymal liver echogenicity, mimicking fatty liver disease (Figure 1) [18, 19]. A prominent parenchymal inhomogeneity with coarsening pattern can also be found, suggesting an irregular patchy infiltration of the parenchyma by confluent granulomas, associated with various degrees of fibrosis surrounding the coalescing tissue (Figure 2) [20–22]. Hepatic nodules usually appear on US as multiple, discrete, and rounded hypoechoic lesions of various sizes. They may mimic liver cirrhosis or nodular regenerative hyperplasia [23]. They can also manifest as areas of increased or similar echogenicity with respect to the adjacent parenchyma, though these patterns are less frequently reported in the literature [17, 24]. Isoechoic lesions can be missed on conventional US and are found on imaging such as CT or MRI. Usually, the nodules are multiple, have different sizes ranging from 1 to 2 mm to several centimeters, are not associated with mass effect on the surrounding parenchyma, and show hypovascularity on Color Doppler US (Figure 2) [17, 22]. Less frequently, single hypoechoic nodules can be observed, raising problems of differential diagnosis with other focal lesions (Figure 1). In our experience enlarged perihepatic lymph nodes can be encountered in almost all patients with circumscribed focal sarcoid liver infiltration.

**Figure 3:**
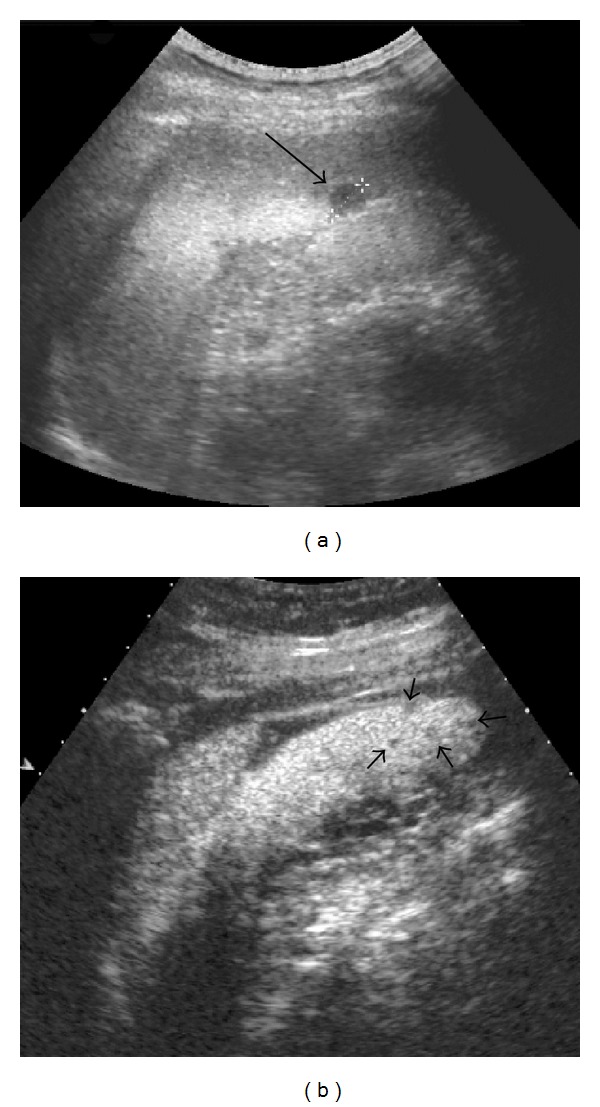
A 53-year-old male with history of pulmonary sarcoidosis. (a) B-mode US showed a rounded and hypoechoic lesion located in the lower pole of the spleen; the nodule did not show flow on Color Doppler US and had a maximum size of 1 cm (arrow, caliper 1). (b) Contrast-enhanced US confirmed the lesion and showed other progressively hypoenhancing nodules of few mm (in median 5 mm, arrowheads) that were not evident on conventional US. Histopathological examination of the spleen revealed infiltration by sarcoidosis.

Splenomegaly can be observed in either absence or presence of focal splenic lesions, and there is a different prevalence in observing discrete nodules among published studies (6–33%), perhaps reflecting ethnic differences in study populations [17]. Splenic nodules usually appear as multiple and hypoechoic focal lesions; they show different size on US (usually less than 10 mm but larger lesions may occur) and are hypovascular on Color Doppler US (Figure 3) [10]. The nodules can also manifest as hyper or isoechoic lesions with respect to the healthy parenchyma. The different patterns can be related to a different degree of fibrosis in the granulomatous tissue [25].

**Figure 4:**
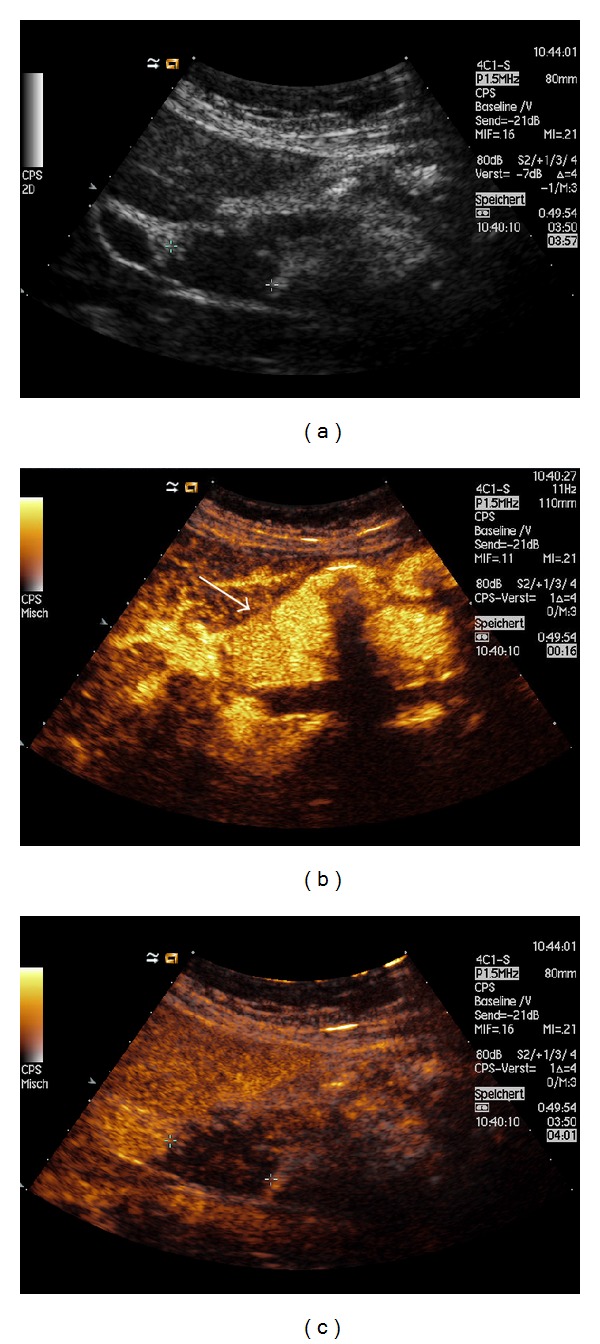
A 64-year-old female with sarcoidosis. (a) B-mode US documented typical prominent perihepatic lymphadenopathy (maximum size of 3 cm, caliper). Contrast-enhanced US showed (b) homogenous enhancement during the arterial phase (arrow) and (c) prominent wash-out (caliper).

Furthermore, enlarged lymph nodes have been observed in up to 76% of cases, both in hepatic and splenic sarcoidosis, and they appear as single or multiple hypoechoic masses that are located most often in periportal, celiac, paracaval, and paraaortic compartments, with sizes between 1 and 2 cm [26]. We generally observed larger perihepatic lymph nodes in advanced liver disease with a lymph node size up to 4–6 cm (Figure 4). In the context of benign diseases such large perihepatic lymph nodes have been observed only in primary biliary cirrhosis (PBC) [27].

Involved abdominal lymph nodes may show inhomogeneous echotexture, with multiple low-level echoes inside [22, 28]. The concomitant enlargement of perihepatic and mediastinal lymph nodes is typical for sarcoidosis but has also been observed in chronic virus hepatitis C [29]. Other not commonly observed findings are represented by punctate calcifications that appear as multiple, small, hyperechoic areas of few millimeters. They can be found in both liver and spleen [20, 26].

Hepatic and splenic involvement by sarcoidosis can be associated with systemic disease or can be isolated. In the latter, the diagnosis is difficult if based only on imaging studies and requires often a biopsy and a histopathological examination of the organs [25–33].

US can also demonstrate some atypical pattern, rarely described in the literature. Some nodules, due to their confluence tendency and irregular appearance, raise the problem of differential diagnosis with neoplastic disorders [10, 34, 35].

Bauones et al. have recently reported a case of incidental finding of multiple hypoechoic and nonvascular splenic nodules that were associated with a significant retraction of the overlying splenic capsule; splenomegaly was not found and no other organ involvement was documented. This atypical finding has mimicked neoplastic disease, and the patient underwent a laparoscopic splenectomy in order to exclude malignancy [25]. In these cases, histopathological examination is required to avoid a misdiagnosis that can lead to a completely different therapeutic approach.

The diagnosis of sarcoidosis can be suspected on the basis of typical clinical, laboratory, and imaging features but is usually achieved with histopathological findings that confirm the presence of noncaseating granulomas and exclude other causes of granulomatous inflammation [1, 36]. Effort should be made to obtain a sample to analyze from biopsy specimen [1, 37–39].

### 3.2. The Role of Computed Tomography and Magnetic Resonance Imaging

Other imaging techniques, such as contrast-enhanced CT (CECT) and MRI, are reliable to evaluate the organ involvement in sarcoidosis. CT can confirm hepatosplenomegaly, and, in most cases, the liver appears homogeneous; sometimes, however, the liver appears heterogeneous and a septa-like pattern can be found after contrast agent injection [17]. CECT can be useful to confirm hepatosplenic nodules or to reveal them, for the first time, after a negative US examination. The lesions manifest as hypodense masses relative to the adjacent healthy tissue, without peripheral enhancement [17, 40, 41]. MRI can also serve as an adjunctive diagnostic tool to confirm the presence of both hepatic and splenic nodules that appear hypointense, relative to the adjacent parenchyma on all sequences, without substantial contrast enhancement after gadolinium administration, and appear less evident on delayed imaging, suggesting equilibration. The nodules are best visualized on T2-weighted fat-suppressed and early-phase dynamic contrast-enhanced images [42]. Furthermore, MRI can be useful to reveal nonspecific hepatic findings such as periportal hyperintensity on T2-weighted images; some authors have suggested that this sign could be associated with a greater tendency of granulomas to localize within periportal spaces [17, 22]. Finally, both CT and MRI can be useful to reveal the presence of punctate calcifications and/or lymphadenopathy [26].

## 4. Contrast-Enhanced Ultrasound (CEUS) in the Assessment of Hepatosplenic Lesions Derived from Sarcoidosis

### 4.1. The Evolving Role of CEUS

In recent years, the use of ultrasound contrast agents (UCAs) has rapidly increased in the clinical practice. Since the first guidelines regarding the use of CEUS in the assessment of liver lesions, released by European Federation of Societies for Ultrasound in Medicine and Biology (EFSUMB) in 2004 and lastly updated in 2013 [43–45], new fields have been investigated with the evaluation of other organs such as spleen, pancreas, gastrointestinal tract, kidneys, and lungs. EFSUMB released an extensive update on nonhepatic use of CEUS, highlighting the wide range of clinical applications that can be carried out [46]. Comments on the guidelines have been published as well [47, 48]. UCAs perform as blood pool tracers and are constituted by gas surrounded by a membrane that prolongs their half-life and provides stability. The envelope consists of organic materials such as galactose, palmitic acid, albumin, and phospholipids. After intravenous injection, enhancement patterns can be evaluated in real time with a higher temporal resolution than in other imaging techniques [44]. UCAs are generally safe and have a low incidence of side effects, without heart, liver, and renal toxicity. Incidence of life-threatening anaphylactoid reactions is very low (0.001% among the 23,000 patients examined) and it is not necessary to perform laboratory tests before starting CEUS examination [45].

### 4.2. CEUS in the Differentiation between Benign and Malignant Focal Hepatosplenic Lesions

CEUS has demonstrated a high overall diagnostic accuracy in the differential diagnosis of focal liver lesions, with similar values of sensitivity and specificity as compared to conventional imaging, such as CT or MRI [49–55]. A recent systematic review and cost-effectiveness analysis found that the pooled estimates of sensitivity and specificity to detect and/or characterize malignant lesions were 95.1% and 93.8% using CEUS, and 94.6% and 93.1% using CECT, respectively [53]. Similar results were obtained by also comparing CEUS and MRI [50]. The use of CEUS is effective in the workup of patients with focal liver lesions, by identifying specific patterns and selecting those who need further diagnostic investigation [56, 57].

Furthermore, several authors have demonstrated that CEUS can provide valuable information in the differential diagnosis of focal splenic lesions with high accuracy [58–62]; Yu et al. have found that the sensitivity, specificity, and accuracy of CEUS in the diagnosis of focal splenic lesions were 91.1%, 95.0%, and 92.0%, respectively. Lower values were obtained using conventional US (75.0%, 84.2%, and 77.3%, resp.) [59]. CEUS can also improve the differentiation between benign vascular and malignant lesions [63] and can be useful when there are no suggestive findings on benign conventional US [64]. The good safety profile, real time evaluation, and absence of radiation exposure are some of the reasons for the wide diffusion of CEUS in the last few years and for the establishment of appropriate indications for its use.

### 4.3. CEUS Patterns of Hepatosplenic Sarcoidosis

Although there is increasing evidence regarding the usefulness and reliability of CEUS, a broad group of disorders have not been investigated so far with this technique. Actually, there is a lack of ad hoc studies in patients with hepatosplenic sarcoidosis, and most evidence derives from description of single case reports or from findings of small case series [10]. Most of the trials have been conducted with the aim to differentiate benign focal lesions from malignant focal lesions, as discussed above.

It is reasonable to expect this lack of data, first of all because sarcoidosis is an uncommon disease, and demonstration of liver and spleen involvement on imaging is even rarer; then, in most of cases, hepatosplenic sarcoidosis appears homogenous on US without evidence of discrete nodules, and second imaging, such as CT and MRI, is preferred to assess the organ involvement in these cases. However, CEUS has documented accuracy to characterize splenic and hepatic parenchymal inhomogeneity, when found [44, 46].

Even if the evidence is limited, hypoechoic hepatic lesions derived from sarcoidosis appear, after UCA injection, as variably arterial enhancing and progressively hypoenhancing nodules in the portal-venous and late phases [10, 65].

**Figure 5:**
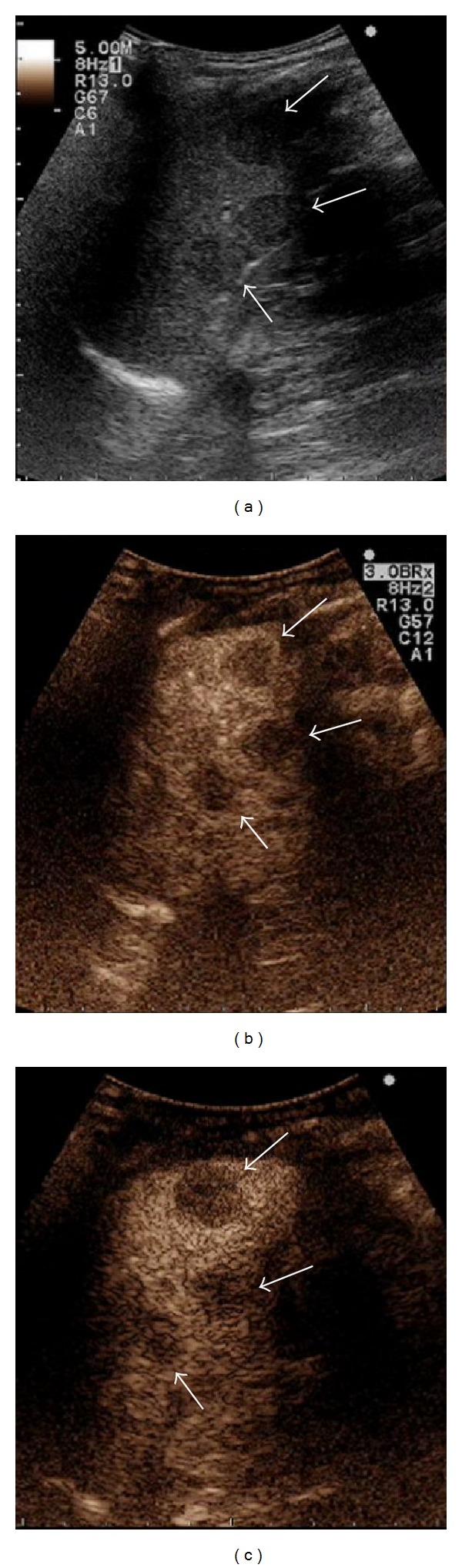
A 45-year-old woman with history of colon cancer, polycystic ovary syndrome, and migraine, who presented with fatigue, weight loss, and headache. No changes in bowel habits were reported. Physical examination revealed only laterocervical lymphadenopathy. (a) B-mode US documented splenomegaly, with parenchyma subverted by multiple and rounded hypoechoic lesions (arrows). The nodules had maximum size of 22 mm and showed no flow on Color Doppler US. Contrast-enhanced US documented (b) rim-like enhancement of the lesions in the arterial phase (7 seconds, arrows) and hypoenhancement in the parenchymal (c) (1 min 20 sec, arrows) and late phases. In view of the patient history, this pattern was first suggestive of malignancy. However, other organs were normal on second imaging, and histopathological examination revealed noncaseating granulomas, suggesting the diagnosis of splenic sarcoidosis (reprinted with permission from [10]).

Also hypoechoic lesions of the spleen appear most often as progressive hypoenhancing nodules, in arterial and parenchymal phases, compared to adjacent splenic tissue, with increasing lesion-to-parenchyma contrast diffusion while moving to parenchymal phase (Figure 3) [58, 65]. The pattern of slight enhancement can be diffusely homogenous or heterogeneous in the arterial phase and diffusely homogenous or dotted in parenchymal phase. Furthermore, some peripheral irregular vessels can be found [58]. Other authors have described a complete absence of enhancement in both arterial and parenchymal phases [66]. In one case, we observed a more rim-like enhancement in the arterial phase, followed by hypoenhancement in parenchymal and late phases (Figure 5) [10, 67]. This pattern can overlap with those observed in neoplastic disorders [57], and biopsy with histopathological examination is, therefore, required to exclude malignancy.

CEUS can be useful to identify hepatic or splenic isoechoic nodules that are not otherwise evident on conventional US; these lesions appear as progressively hypoenhancing masses (Figure 3) [24]. Sometimes, they appear as almost isoenhancing nodules in the late phase (Figure 2). CEUS can also confirm the presence of abdominal lymph nodes enlargement with homogeneous enhancement, suggesting a benign inflammatory pattern (Figure 4) [68].

## 5. Conclusion: Implications for Clinical Practice and Future Perspectives

The limited evidence regarding CEUS findings in hepatosplenic sarcoidosis raises the need for further studies that evaluate the role of CEUS in this uncommon disease. Although the most observed pattern is characterized by absence or less enhancement of the nodules with respect to the healthy parenchyma, no studies have reported CEUS findings in hyperechoic lesions derived from hepatosplenic sarcoidosis. It would be interesting to explore these patterns and to see if there is a different behavior after UCA administration; however, we expect similar findings for hyperechoic nodules on CEUS to that of hypo- and isoechoic lesions, because of their similar hypodense appearance on CECT [17, 24]. CT and MRI are often preferred to evaluate organ involvement in sarcoidosis, because lesions with similar echogenicity to the healthy parenchyma cannot be found on conventional US. CEUS can overcome these limitations and reveal hepatic and splenic nodules. In our experience, we observed that conventional ultrasound may be also useful to show treatment response and a significant reduction in the size of hepatosplenic lesions after steroid therapy. Further studies should evaluate any change of contrast enhancement pattern after treatment of focal lesions and perihepatic lymphadenopathy [27]. CEUS could be useful to follow up the lesions over time, thus avoiding unnecessary radiation exposure associated with CT imaging and kidney damage in patients at risk, after administration of iodine contrast or gadolinium.

In conclusion, hepatosplenic sarcoidosis remains so far a challenging diagnosis [69, 70]. Imaging findings are often nonspecific, and, in cases of isolated abdominal organ involvement, a diagnosis of sarcoidosis can be achieved only by revealing noncaseating granulomas in tissue specimens and excluding other causes of granulomatous inflammation [36, 71, 72]. The role of conventional imaging, such as ultrasound, CT, and MRI, can be reserved in the staging of the disease and not for diagnostic purposes, because isolated hepatosplenic sarcoidosis can be misdiagnosed with disorders such as lymphoma or metastasis that manifest with similar findings and may raise an erroneous suspicion, especially if the patients have a history of malignancy. CEUS has potential in the assessment of hepatosplenic sarcoidosis, but there is need of prospective, controlled trials that aim to explore CEUS patterns in comparison with conventional imaging and biopsy, before drawing definite conclusions.
